# Atraumatic Splenic Rupture Associated With Epstein-Barr Virus: A Case Report

**DOI:** 10.7759/cureus.87030

**Published:** 2025-06-30

**Authors:** Daniel Alejandro Gamón Briseño, Ana Lucia Luna Sada, Nora Lis Flores Olmos, Rafael Delgado Duarte, Victor Mario Ortega Valerio

**Affiliations:** 1 Department of General Surgery, Hospital General Instituto de Seguridad y Servicios Sociales de los Trabajadores del Estado (ISSSTE) Zacatecas, Zacatecas, MEX; 2 Department of Internal Medicine, Hospital General Instituto de Seguridad y Servicios Sociales de los Trabajadores del Estado (ISSSTE) Zacatecas, Zacatecas, MEX; 3 Department of General Surgery, Hospital Regional Valentín Gómez Farías, Zapopan, MEX

**Keywords:** acute abdomen, epstein-barr virus infections, spleen, splenectomy, splenic rupture

## Abstract

Infectious mononucleosis, caused by Epstein-Barr virus (EBV), is typically self-limiting but can lead to rare, life-threatening complications such as atraumatic splenic rupture (ASR). We report the case of a 16-year-old female presenting with left upper quadrant pain and peritoneal signs. The initial laboratory findings revealed grade I anemia and leukocytosis. Images obtained by simple CT revealed the presence of free intra-abdominal fluid and a perisplenic hematoma. This raised the diagnostic suspicion of splenic rupture in the context of an acute EBV infection. Emergency splenectomy was required due to hemodynamic instability. The patient recovered well postoperatively. ASR, though uncommon, demands prompt recognition and management in mononucleosis patients with abdominal symptoms and potential circulatory compromise.

## Introduction

Atraumatic splenic rupture (ASR) is a rare but potentially life-threatening condition, most commonly associated with infectious diseases, especially infectious mononucleosis (IM) caused by Epstein-Barr virus (EBV), as well as medical procedures and hematologic disorders [1]. The characteristics of splenic rupture and infarction related to IM remain underinvestigated in recent years, with most available data coming from case reports [2].

Splenic rupture is a rare complication of IM, occurring in 1-2 per 1,000 cases [3]. Also known as glandular fever, IM is typically caused by primary EBV infection. While often asymptomatic in children, it usually presents in adolescents and young adults as a mild, self-limiting syndrome [3-5]. EBV, a double-stranded DNA virus of the herpesvirus family, is primarily transmitted via saliva, hence its nickname “kissing virus,” and accounts for nearly 90% of IM cases [6-8]. It is estimated that over 90% of adults worldwide have been infected [6].

Following a 4-8-week incubation period, symptoms may include fever, pharyngitis, lymphadenopathy, malaise, fatigue, and splenomegaly in up to 65% of cases [7]. Rare complications include myocarditis, pericarditis, pancreatitis, and splenic rupture, which occurs in 0.1% to 0.5% of patients [5,9]. The first case of spontaneous splenic rupture in IM was described in 1941 [10]. Since then, around 100 cases have been reported, mostly in adolescents and young adults [4,10]. Up to 90% of patients are under 30 years old, with an average age of 22 years and a male predominance of 70% [4].

Splenic rupture results from progressive enlargement due to EBV-induced lymphocyte proliferation, leading to parenchymal changes and increased susceptibility to rupture [4].

## Case presentation

A previously healthy 16-year-old female presented to the emergency department with acute abdominal pain, vomiting, diarrhea, and general symptoms lasting 24 hours. Five days before admission, she developed erythematous pharynx and right posterior cervical lymphadenopathy. She received outpatient treatment under the suspicion of viral gastroenteritis. Twenty-four hours later, she returned to the emergency department with acute abdominal pain accompanied by diaphoresis and tachycardia. While in the emergency room waiting area, she experienced a near-syncope episode. Vital signs were obtained, revealing hemodynamic instability.

Physical examination of the abdomen showed generalized pain, predominantly in the left upper quadrant, with muscle guarding, hyperesthesia, and hyperalgesia. Laboratory results (Table 1) revealed grade I normocytic anemia, leukocytosis, and elevated aspartate aminotransferase, alanine aminotransferase, and alkaline phosphatase.

**Table 1:** Patient’s admission laboratory findings in the emergency room.

Parameter	Value	Reference range
Red blood cells	3.72 mill/µL	4.2–5.4 mill/µL
Hemoglobin	10.4 g/dL	12–16 g/dL
Mean corpuscular volume	88.4 fL	82–98 fL
Hematocrit	32.90%	38–47%
Platelets	150 µL	150–400 µL
Leukocytes	12.88 cells/µL	5–10 cells/µL
Neutrophils	48.60%	40–70%
Prothrombin time	14 seconds	11-3–15.9 seconds
International normalized ratio	1.05 seconds	0.8–1.2 seconds
Partial thromboplastin time	27.4 seconds	25.10–37.5 seconds
Sodium	141 mmol/L	135–145 mmol/L
Potassium	3.8 mmol/L	3–4.5 mmol/L
Chlorine	111 mmol/L	98–107 mmol/L
Albumin	4.2 g/dL	3.5–5 g/dL
Aspartate aminotransferase	162 U/L	4–32 U/L
Alanine aminotransferase	321 U/L	30–65 U/L
Alkaline phosphatase	282 U/L	38–126 U/L
Glucose	118 mg/dL	70–100 mg/dL
Urea nitrogen	10 mg/dL	8–23 mg/dL
Urea	20.6 mg/dL	10–50 mg/dL
Creatinine	0.8 mg/dL	0.6–1.3 mg/dL

**Figure 1:**
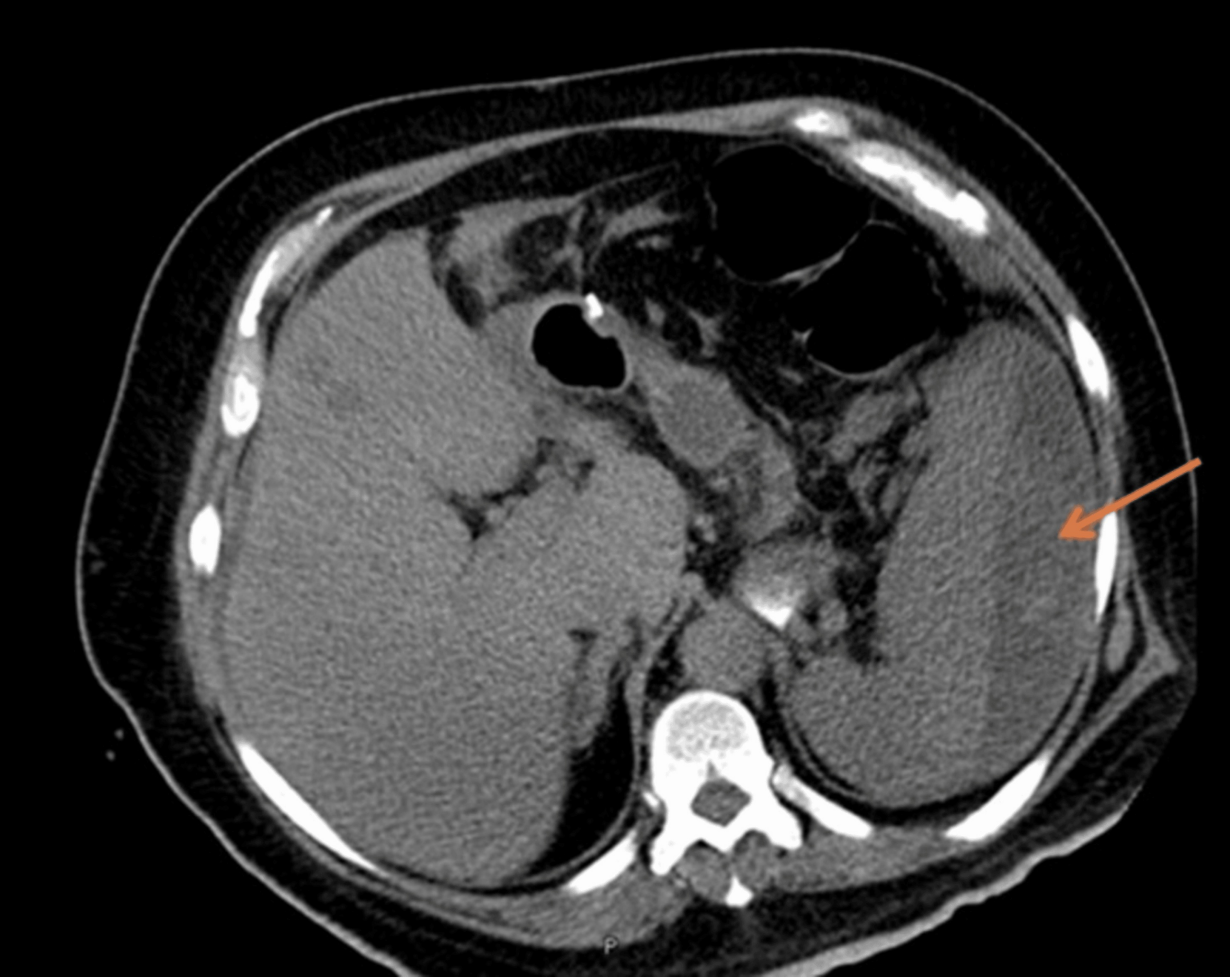
Non-contrast axial CT scan. The arrow is showing a hypodense area in the splenic region consistent with a perisplenic hematoma.

A non-contrast abdominal CT (Figure 1) scan revealed free fluid in the abdominal cavity and perisplenic hematoma.

Given the patient’s condition, she was taken to the operating room for exploratory laparotomy. Upon entering the abdominal cavity, 500 cc of hemoperitoneum was evacuated. Exploration of the abdominal quadrants showed no abnormalities in the liver, colon, pancreas, small intestine, or adnexa. Upon accessing the spleen, an ischemic patch, active bleeding, and a ruptured capsule were observed. Eight surgical pads were placed for hemostasis, followed by splenectomy. The specimen was sent to the hospital’s pathology department. The patient remained under observation for three days and was discharged after clinical improvement.

**Figure 2:**
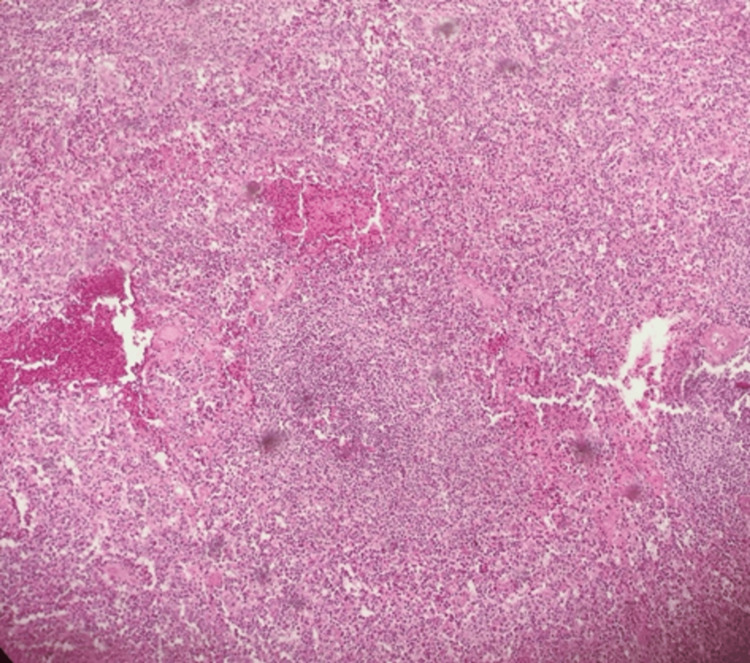
Histological slide of the spleen at 10× magnification showing recent hemorrhage in the splenic parenchyma with hematoma formation and an area of necrosis with ischemia.

At her follow-up visit, histopathological results (Figure 2) and EBV serology (Table 2) were available and revealed a recent EBV infection in a possible resolving or late active phase. These findings confirmed atraumatic splenic rupture associated with acute Epstein-Barr virus infection.

**Table 2:** Epstein-Barr virus (EBV) serology.

Parameter	Value	Reference range
Heterophile antibodies	Negative	Negative
Anti-EBV early antigen Ig	14 U/mL	<10
EBV capsid IgG	65.3 U/mL	<20
EBV capsid IgM	>160 U/mL	<40
EBV nuclear antigen IgG	16.10 U/mL	<5

## Discussion

ASR can be caused by various conditions, including neoplastic disorders, infectious diseases, inflammatory disorders, iatrogenic or pharmacological causes, mechanical trauma, and idiopathic origins. Neoplasms are the most common etiology, accounting for up to 30.3%. Infectious disorders are the second most common cause, representing up to 27.3%, and include hepatitis A virus, HIV, cytomegalovirus, dengue virus, and EBV, the latter being the most frequent cause of ASR [11].

The overall incidence of ASR is estimated at approximately 3% of all splenic ruptures, with a mortality rate of 9%, which is lower than the 14% associated with traumatic rupture [4,6,11]. A literature review across various databases from 1970 to 2022 identified a total of 186 cases of splenic rupture associated with EBV. It showed a higher incidence between the ages of 12 and 22 years, with an average age of 22 years and a male predominance of 70%, or a 3:1 male-to-female ratio [2,6,12]. In the context of EBV-associated ASR, the reported incidence is between 0.1% and 0.5%, occurring most commonly within the first four weeks of symptom onset, though it may extend up to eight weeks [3,11,13].

Splenic rupture is believed to result from splenomegaly caused by lymphocytic infiltration, stretching of the splenic capsule, and weakening of the trabeculae. These factors, combined with splenomegaly and reduced protection from the thoracic cage, increase the risk of rupture [12].

The virus enters B lymphocytes after the interaction of the viral glycoprotein gp350 with the CD21 receptor on the B-cell surface. The virus enters the human body through the mucosa at the level of the tonsils and infects B lymphocytes in the underlying lymphoid tissue. Eventually, latently infected B cells enter peripheral circulation as memory cells that do not express viral proteins and are thus invisible to the immune response. When these cells return to the Waldeyer’s ring, they occasionally differentiate into plasma cells, triggering viral replication and making B cells vulnerable to immune attack. The proliferation of B cells and viral replication are controlled by a robust CD8+ cytotoxic T-cell-mediated immune response, which is responsible for most of the symptoms [4,14].

In EBV infection, the spleen increases in volume and fragility due to extensive infiltration by lymphocytes and monocytes, making it susceptible to spontaneous rupture. The cause of rupture is likely a combination of acute increases in portal pressure and sudden intra-abdominal pressure due to coughing, sneezing, straining, turning in bed, or defecating [13].

Ultimately, the spleen may rupture due to increased fragility and/or minor physical events that elevate intra-abdominal pressure [15], through the following mechanisms: (1) increased intrasplenic pressure caused by cellular or reticuloendothelial hyperplasia with engorgement and vascular occlusion; (2) compression from increased intra-abdominal pressure during physiological activities (e.g., coughing, sneezing, defecation); and (3) vascular occlusion caused by reticular endothelial hyperplasia leading to thrombosis and infarction [16].

The classical triad of symptoms in IM includes a 4-5-day prodrome, followed by pharyngitis, fever, lymphadenopathy, and splenomegaly, in addition to headache, malaise, and fatigue [3,4,6]. Pharyngitis is a very common component of IM and typically presents acutely with moderate pain and tonsillar exudates of a grayish-yellow appearance. Lymphadenopathy primarily affects the posterior cervical chain and is an early disease marker [7]. Splenomegaly is detected in 15% to 65% of IM cases, and the spleen can enlarge up to four times its normal size [3]. This finding has a sensitivity of 7% and a specificity of 99% [6].

The most frequent symptom in splenic rupture is acute abdominal pain localized to the left upper quadrant, reported in up to 88% of cases [6,9]. The pain may radiate to the left shoulder tip, known as Kehr’s sign, due to diaphragmatic irritation from hemorrhage. This sign occurs in 17% to 50% of cases and is explained by the phrenic nerve, which innervates the diaphragm and shares roots (C3 and C4) with the supraclavicular nerves. Another indicative sign of splenic rupture is Ballance’s sign, described as a palpable mass in the left hypochondrium [11]. Other clinical signs suggesting splenic rupture include hemodynamic instability, peritoneal irritation, and abdominal muscle guarding.

Ultrasound is frequently used as the initial imaging modality for suspected splenic rupture, while contrast-enhanced CT or MRI are the current diagnostic standards [3]. Ultrasound findings include splenomegaly, hypoechoic areas in the splenic parenchyma, signs of splenic fracture, and hemoperitoneum. Subcapsular fluid may also be seen, though perisplenic clots may initially be indistinguishable from the rest of the spleen due to their similar echogenicity; in addition, this modality allows evaluation of splenic vascularization [4]. On CT, typical signs of splenic rupture include hyperdense or hypodense foci in the spleen, with 35-60 Hounsfield units (HU) in active bleeding and 60-80 HU in later stages due to clotting, along with intracapsular or intraperitoneal fluid [17]. Lacerations, fractures, or splenic parenchymal disruption and hemoperitoneum may be observed. In atraumatic ruptures, the most frequent sign is the perisplenic hematoma, which may vary in density depending on the age of the clot. In acute stages, the hematoma appears hyperdense and is referred to as the “sentinel clot” sign [4].

For etiologic diagnosis of splenic rupture, various serologic tests are available, such as the Monospot test and EBV-specific antibodies. The Monospot test is a specific diagnostic test for EBV infection. It detects heterophile antibodies via latex agglutination or red blood cell assays. It has a sensitivity of 81% to 95% and a specificity of 98% to 100%. A positive result strongly suggests infection, but a negative result does not rule it out. It is important to note that over 25% of cases test false negative within the first week, as heterophile antibody titers may take 2-5 weeks to reach detectable levels [6,7]. The main antibodies analyzed include IgM and IgG against the viral capsid antigen (VCA), IgG against the nuclear antigen (EBNA), and IgG against the early antigen (EA). IgM and IgG against EBV-VCA are typically present in the acute phase and are diagnostically valuable during this stage. Additional tests for EBNA antibodies can help differentiate between acute and past infections. VCA and EBNA antibody tests offer greater sensitivity and specificity but are more expensive and time-consuming [7,8].

Due to the rarity of splenic rupture associated with mononucleosis, no clear consensus exists on treatment strategy [2]. Management of spontaneous splenic rupture in IM is similar to that of traumatic splenic rupture [13]. Traditionally, total splenectomy was performed, but the risk of post-splenectomy infection has increased interest in spleen-preserving approaches and conservative management [11]. However, surgical treatment with splenectomy remains necessary in patients with hemodynamic instability despite resuscitation and radiologic evidence of splenic injury on contrast-enhanced CT [12]. Numerous case reports document successful conservative management of splenic rupture under close clinical observation and follow-up with imaging, mainly ultrasound and CT [18]. Literature reports success rates of up to 80% for conservative management, with mortality as low as 4.4% [11]. In recent years, conservative approaches have included interventional radiology techniques such as splenic artery embolization or partial splenectomy [4,11]. It is important to consider that after splenectomy, vaccination against *Streptococcus pneumoniae*, *Haemophilus influenzae*, and *Neisseria meningitidis* is required [19].

## Conclusions

IM caused by EBV is a common condition, especially among adolescents and young adults. Although it generally has a favorable and self-limiting course, it can be associated with potentially fatal complications, such as ASR. Clinicians should be aware of this rare complication, given the potential risk of diagnostic delay. Clinical suspicion should be raised in patients with mononucleosis who present with sudden abdominal pain, signs of peritoneal irritation, or hemodynamic instability. Imaging studies, especially contrast-enhanced CT, play a crucial role in diagnosis. Management depends on the patient’s hemodynamic status. Conservative treatment is increasingly used in hemodynamically stable patients, while splenectomy remains indicated in those with instability or active bleeding. In patients undergoing splenectomy, prevention against encapsulated microorganisms should be taken into account through pneumococcal vaccination, meningococcal vaccination, and *Haemophilus influenzae* type b vaccination. ASR should always be considered as a differential diagnosis in patients with an acute abdomen and a recent history of mononucleosis, especially if splenomegaly is present.
